# Associations between Pretend Play, Psychological Functioning and Coping Strategies in Pediatric Chronic Diseases: A Cross-Illness Study

**DOI:** 10.3390/ijerph17124364

**Published:** 2020-06-18

**Authors:** Daniela Di Riso, Elena Cambrisi, Simone Bertini, Marina Miscioscia

**Affiliations:** 1 Department of Developmental Psychology and Socialization, University of Padova, 35121 Padova, Italy; daniela.diriso@unipd.it (D.D.R.); elena.cambrisi@studenti.unipd.it (E.C.); simone.bertini@studenti.unipd.it (S.B.); 2 Department of Women’s and Children’s Health, Hospital-University of Padua, 35128 Padova, Italy

**Keywords:** type 1 diabetes mellitus (T1DM), cystic fibrosis, leukemia, Affect in Play Scale—Brief (APS-Br), coping, psychological adjustment, separation anxiety

## Abstract

Children with chronic illnesses are called to undertake complicated processes of adjustment and re-organization in their daily lives; as a result, they could experience several internalizing problems. Symbolic play could be a useful way to cope with these difficulties. The main aim of this paper is to assess pretend play, coping, and psychological symptoms in three groups of school-aged children with pediatric chronic diseases. The study involved 44 Italian school-aged, chronically ill children: 16 with type 1 diabetes mellitus (T1DM), 12 with cystic fibrosis (CF), and 15 with Leukemia. All patients were assessed by the Affect in Play Scale–Brief version (APS-Br), and the Children’s Coping Strategies Checklist–Revision1 (CCSC-R1). Children with T1DM and CF also completed the Separation Anxiety Symptom Inventory for Children (SASI-C) and the Strengths and Difficulties Questionnaire (SDQ)–children’s version. Cohen’s d (effect size) was applied between clinical and normative samples, and it showed a more organized play (APS-BR), but a more negative affect tone, comfort, and frequency of affect expression. Comparing APS-BR and CCSC-R1 rates between the three groups, significant differences were found for all the APS-BR dimensions, except for tone, and for CCSC-R1 seeking understanding. Comparing SASI-C score between T1DM and CF, higher scores were found for children with CF. In the end, correlations between all dimensions highlighted several relationships between play, coping, and adjustment problems for children with T1DM, and relationship between affect play and all variables for children with CF. Symbolic play helps chronically ill children to express emotions; helping them, as well as clinicians, to understand the difficulties caused by chronic conditions, and to cope with them.

## 1. Introduction

The term “chronic disease” indicates a condition that lasts three months or more, that involves medical care constantly, and/or limits activities in daily lives [1]. The prevalence rates of pediatric chronic conditions range from 13% to 27% [2]; due to scientific progress, many children diagnosed with chronic diseases considered serious and lethal, such as cystic fibrosis or muscular dystrophies, are now living into adulthood [3]. 

Among the different types of chronic diseases, the present paper focused on three pediatric chronic illnesses: an autoimmune disease that can occur at any time (type 1 diabetes mellitus), an inherited genetic disease (cystic fibrosis), and a genetic disease that, in most cases, is not hereditary (leukemia).

Type 1 diabetes mellitus (T1DM) is one of the most common chronic endocrine illness affecting young people in Italy. Epidemiological data report that in Italy, 20,000 children showed T1DM diagnosis, with the most frequent onset between 9 to 11 years old [4]. T1DM requires self-care practices, as well as invasive, such as insulin administration and blood glucose monitoring. T1DM children are often involved in strict diet regimens, sports activities, and hospitalization periods. Cystic fibrosis (CF) is a common life-shortening disease that causes severe damage to the lungs, digestive system, and other organs in the body. In this condition, a defective gene affects cells that produce secreted fluids (mucus, sweat, and digestive juices). In Italy, the prevalence rate of CF was 8.8/100.000 individuals in 2016, and mainly affects the age group of 7–35 years [5]. Leukemia is a cancer of the blood or bone marrow that can develop due to a problem with blood cell production; it is the most common cancer in subjects under 15 years of age. Acute leukemia develops quickly and worsens rapidly, but chronic leukemia gets worse over time. A study by Francisci and colleagues [6] found that cancers diagnoses in children under 15 years of age represent 1.2% of all cancer cases in Italy; the survival rate has greatly improved over the past 40 years in all high-income countries.

In these chronic conditions, children have to adjust to important changes in their everyday lives; physical limitations often lead children to psychosocial restrictions [7]. Depending on the condition, chronically ill children may experience frequent hospitalizations, painful diagnostics and treatment processes, fear of death, and prolonged separation from family and friends that lead to social exclusion, internalizing disorders [8,9,10], difficulty with peers [11,12], and externalizing problems [13,14,15].

The literature clearly show how children with CF, T1DM, and leukemia experience a varying rate of depressive, stress, and anxiety symptoms [16,17,18,19]. Besides experiencing frequent hospitalizations, chronically ill children may need close monitoring to avoid possible medical crisis, which could lead to severe high risks for health, including death. Consequentially, they could feel more anxious about being away from home or their parents, and might be more prone to developing separation anxiety symptoms compared to healthy peers [20].

Adjustment to chronic illness depending on various aspects, such as environmental variables [21], parental and social support or intelligence [22]; psychological factors, such as resilience or coping strategies [23] play a significant role too in mediating the effects of diseases-related distress [24]. Between the variety of stressors that pediatric patients may experience, there are controllable situations (e.g., boring hospital stays) or un-controllable situations (e.g., treatment side-effects); these aspects require specific coping strategies in chronically ill children [25,26]. In children with T1DM, active coping and perception of control seem to be related to better metabolic control [27]; avoidance coping strategies are connected to worse metabolic outcomes and poor treatment compliance [28]. Ayers and colleagues [29] pointed out that CF children showed coping strategies to increase control and to avoid stressful situations, such as not looking or not thinking about frequent invasive procedures they would need to undergo. A study by Han, Liu and Xiao [30] found that primary school-aged children with leukemia use mainly Problem-Focused Coping (PFC), cognitive strategies that actively seek information to decrease anxiety and fear about the illness and treatment. Failo et al. [31] examined the relationships between coping strategies in a sample of three disease cohorts: oncological diseases, rheumatic diseases, and CF. Similar coping strategies for pain management were reported across the three disease cohorts (cognitive coping, problem-solving, social support, distraction). Children with cancer reported lower adaptive coping strategies than rheumatic children but similar coping profiles in respect of CF children. 

Interventions enhancing coping skills and emotion expression have shown a positive effect on quality of life for chronically ill children [32]. Pretend play is one of the means through which children could express emotions [33]. Rae and Sullivan [34] highlighted that play interventions are effective for decreasing hospital-related fears, preventing anxiety from worsening, improving self-esteem, and medical regimen compliance. Moore and Russ [35] confirmed play utility as a way to prevent negative psychosocial outcomes in medical settings, but highlighted that literature referred to contexts widely different, in terms of setting or children’s medical conditions. Nevertheless, assessment of play seemed to be effective in detecting specific patterns, highlighting both strengths and weaknesses in play skills in the clinical sample [36,37,38]. Most of the studies included hospitalized children scheduled for different surgery procedures (e.g., minor surgery, invasive dental procedures, tonsillectomy) and few studies investigated the use of play for pediatric chronic samples. Symbolic play was assessed in children with T1DM and asthma, reporting its usefulness in increasing chronic condition adaptation and the child’s ability to cope in daily challenges [39]. Gariepy and Howe [40] examined the association between perceived distress and play skills in children with leukemia, showing that their play was less frequent, less structured, and included a narrower variety of themes than the control group. Recently, Nabors and Liddle [41] examined the perceptions of a small group of children with various chronic illnesses, with a play interview (through the means of small toys) recalling the hospital figures and environment. Results indicated that patients were able to express resilience, such as adaptive coping strategies, and difficulties related to their health issues, such as worries and distress. As far as we know, no study has focused on the comparison between T1DM, CF, and leukemic children in respect to psychological variables. 

The main goal of the present paper is to assess play, coping strategies, and symptoms in three groups of school-aged children affected by leukemia, T1DM, and CF.

1. The first aim is to compare play and coping strategies among leukemia, T1DM, and CF. Evidence suggests that play and coping strategies might show variability across different chronic conditions [31,40].
2. The second specific aim is to assess the differences between T1DM and CF children concerning psychological symptoms. Literature suggests that pediatric chronically ill patients show a prevalence of internalizing symptoms [17,18].
3. The third aim is to assess association between play, coping strategies, and psychological symptoms in T1DM and CF. We expected correlations between play cognitive and affective dimensions, symptoms, and coping [37].

## 2. Materials and Methods 

### 2.1. Participant 

A total of 44 chronically ill children were recruited in pediatric wards in North and Central Italy. Sixteen children with T1DM (6 males; M_age_ = 7.94, SD = 2.17); 13 children with CF, (8 males, M_age_ = 8.00, SD = 1.68); 15 leukemic children, (6 males, M_age_ = 9.00, SD = 1.20). While CF is diagnosed since birth, T1DM was diagnosed between 9 and 96 months before the beginning of this study (M_months_ = 44.94; SD = 27.61). Leukemic children were tested between 2 and 3 months, starting from the diagnosis communication. All of the children had at least one period of hospitalization since the diagnosis, but they were assessed during day hospital. None of the children showed comorbidity with other pediatric conditions, cognitive impairment, or psychiatric symptoms. Family socioeconomic status was evaluated using the Four-Factor Index of Socioeconomic Status (SES) scores by Hollingshead [42]. Ninety-one percent of the families were middle-class, with no differences between groups.

### 2.2. Procedures 

The administration was carried out in line with the ethical standards for research [43]. The Ethical Committee of Padova University approved the study (2017/num 2310). The study was introduced to parents by the researcher in agreement with the medical staff, during scheduled medical visits in day hospital. Parents who agreed to participate signed the informed consent, after reading a detailed informative flyer. Each patient completed administration in a quiet room in the pediatric ward after a brief warm-up conversation (useful for making the patient more comfortable with the researcher). Children were first engaged in the play task to assess cognitive and affective variables of pretend play; 5 min later, the research administrated self-report questionnaires to children. The session lasted 30 min for leukemic children and 50 min for T1DM and CF patients. In order to support children, self-report items were read aloud by the researcher. If required, the researcher might have provided brief (but not suggestive) explanations of some words. Nevertheless, the researcher was previously trained to handle stressful or fatiguing situations. The administrations were scheduled in order to not interfere with medical procedures. No reward was offered for enrollment. 

### 2.3. Measures 

Children with T1DM, CF, and leukemia were assessed with Scale–Brief version and Children’s Coping Strategies Checklist–Revision1. Patients with T1DM and CF also completed the Strengths and Difficulties Questionnaire–children’s version, and the Separation Anxiety Symptom Inventory for Children. 

Affect in Play Scale–Brief version (APS-Br) [44,45] is a 5-min coding play task assessing affective and cognitive dimensions in child’s play. It includes in vivo coding and does not require videotaping. The task requires children to play with a set of plastic and stuffed toys, including animals (e.g., elephant, bear, dog) and objects (e.g., plastic cups and plastic cars). They are asked to play aloud, telling a story involving the material presented. It includes two emotional variables: frequency of affect expression (coded on a 4-point Likert scale, from 1 = low emotional expression, to 4 = high emotional expression) and tone, which measures the proportion of positive and negative affect expressed during the task (coded on a 4-point Likert scale, from 1 = predominately negative affect, to 4 = predominately positive affect). Furthermore, it comprises three cognitive variables coded on a 5-point Likert scale, called organization, imagination, and comfort. APS-Br was validated also with Italian children, showing good psychometric properties [45].

Children’s Coping Strategies Checklist–Revision1 (CCSC-R1) [46] is a self-report questionnaire that includes 54 items. These items describe several types of coping strategies: active coping (e.g., “I do something to make things better”), avoidance coping (e.g., “I listen to music”), and support-seeking coping strategies (e.g., “I tell people how I feel about the problem”). Children have to indicate how frequently they use the coping strategy described in each item on a 4-point Likert scale (1 = never, 2 = sometimes, 3 = often, and 4 = always). This questionnaire is made of 13 subscales, which are divided into 5 dimensions: Problem-Focused Coping, Positive Cognitive Restructuring, Distraction Coping Strategies, Avoidance Coping Strategies, and Support-Seeking Strategies. Italian validation presents good psychometric properties [47].

Strengths and Difficulties Questionnaire–children’s version (SDQ–Children’s version) [48] is a 25 item self-report questionnaire. The SDQ measures 25 behavioral traits (coded on a 3-point Likert scale, 0 = not true, 1 = quite true, and 2 = certainly true) divided into 5 subscales. Four of these subscales allow to assess adjustment difficulties (Emotional Symptoms, such as “I have many fears, I am easily scared” Conduct Problems, Hyperactivity-Inattention, and Peer Problems), and one to assess the prosocial behaviors (Prosocial Behaviors, such as “I am helpful if someone is hurt, upset or feeling ill”). The first four subscales are summed to obtain a Total Difficulties Score. Higher scores indicate more problematic behavioral traits [49]. 

Separation Anxiety Symptom Inventory for Children (SASI-C) [50] is a 15-item self-report questionnaire that includes the same items as the adult retrospective version [51], but written in the present tense. This questionnaire is divided into two subscales: separation anxiety and fears of what can happen in the night. Children rated their anxiety on a 4-point Likert scale (0 = never had this feeling, 1 = this feeling happens occasionally, 2 = this feeling happens fairly often, and 3 = this feeling happens very often). This measure shows good psychometric properties [50,52].

## 3. Results

### 3.1. Differences between T1DM, CF, and Leukemia in Pretend Play and Coping Strategies

Differences in APS-Br dimension scores between clinical and normative samples [45] were calculated using Cohen’s d effect size [53]. Means, standard deviations, and Cohen’s d for all three groups were reported in Table 1. Only results with medium and large effect sizes were interpreted. In respect to cognitive dimensions, organization was higher for T1DM children, while it was lower for children with leukemia in comparison with normative data. Leukemic children also reported low score in imagination. Comfort was lower for both children with CF and Leukemia. As for the affective dimensions, only children with leukemia reported lower score in frequency of affect expression and tone than the normative sample. Cohen’s d effect size (1992) was also used to calculate differences in CCSC-R1 dimension scores between clinical and normative samples [38]. Results were reported in Table 1. Problem-focused coping was lower for children with CF in comparison with normative data. Both children with T1DM and leukemia reported low scores in distraction strategies. 

Differences in APS-Br and CCSC-R1 scores between the three groups were calculated with the Kruskal–Wallis test. Regarding APS-Br scores (see Table 1 for means and SD), children with leukemia reported significantly lower scores (*p* < 0.05) for organization, imagination, comfort and frequency of affect expression in respect with the other groups. As for CCSC-R1, no significant differences were found across the three different groups.

### 3.2. Differences between T1DM, CF, in Psychosocial and Separation Anxiety Symptoms

Differences in SDQ factor scores between T1DM, CF children, and normative sample [49] were calculated using Cohen’s d effect size (1992). Results were reported in Table 2. Only results with medium and large effect sizes were interpreted. Total difficulties score, hyperactivity-inattention, and peer problems were higher than the normative sample for children with T1DM and CF, while the prosocial behaviors score was higher only for T1DM children. Cohen’s d effect size [53] was also used to calculate differences in SASI-C dimension scores between clinical and normative samples [51]. Results were reported in Table 2. Total score and separation anxiety were higher than the normative data for both children with T1DM and CF, while fear of something that can happen in the night was higher only for CF children.

Differences between T1DM and CF groups in SASI-C and SDQ were calculated with the Mann–Whitney U. No significant differences for SDQ were found. Results for SASI-C were reported in Table 3. SASI-C Total scores, separation anxiety, and fears of something that can happen in the night were significantly higher for CF children. 

### 3.3. Correlations between Pretend Play, Coping Strategies, Psychosocial, and Separation Anxiety Symptoms in T1DM and CF

Spearman two tails correlations between APS-Br and CCSC-R1, SDQ, SASI-C were conducted for T1DM and CF separately. For children with T1DM, correlations were conducted while controlling for the time from the diagnosis. Only significant correlations were taken into consideration (*p* < 0.05). As for the affective dimensions, the frequency of affect expression was positively related to two of SDQ dimensions: total distress score (r = 0.589) and emotional symptoms (r = 0.586). Positive correlations were also found between tone and SDQ peer problems (r = 0.561), and CCSC-R1 positive cognitive restructuring (r = 0.550). Other significant correlations were found between imagination and CCSC-R1 positive cognitive restructuring (r = 0.620), and between organization and CCSC-R1 positive cognitive restructuring (r = 0.597). No relations were found between APS-Br and SASI-C. 

For children with CF, positive significant correlations were found between tone and SDQ prosocial behavior (r = 0.709). A negative correlation was found between tone and SASI-C fears of somethings that can happen in the night (r = −0.557). As for cognitive dimensions, significant correlations were found between comfort and SASI-C separation anxiety (r = 0.582). No relations were found between APS-Br and CCSC-R1.

## 4. Discussion

Pediatric chronic diseases could lead to experiences of depression, anxiety, worry, trauma reactions, and feelings of isolation and grief [17,18]. The main aim of the present paper was to evaluate the interaction between pretend play, coping strategies, and symptoms in three different groups of school-age pediatric chronic patients. To the best of our knowledge, no study focused on the comparison between T1DM, CF, and leukemic children. Moreover, evidence suggested that researches including samples with different pediatric conditions mostly compared clinical samples and healthy children, with no specific attention in discussing differences between clinical sub-samples [7,8,9,11,12,13,14,15,16,17,18,19,20].

In respect to the first aim, leukemic children showed the greatest impairment in play-skills compared to normative sample, T1DM and CF children, related to both cognitive and affective dimensions. This is consistent with literature suggesting that leukemic children present a more compromised ability to play [40]. Moreover, leukemic children enrolled in the present paper had their diagnosis communication very recently in respect to the other clinical groups. Presumably, they might still struggle with the adaptation process to their new and severe medical condition, including debilitating medical procedures that have already begun. On the other hand, T1DM and CF children showed a general healthy pattern in cognitive and affective play skills. CF children confirmed this pattern, but they just presented poorer comfort during the play session, maybe due to their respiration fatigue, which could have interfered with the request to tell a story aloud involving the material presented. Moreover, T1DM scored higher on the capacity to organize a structured play scenario in respect to normative data. This data partially confirmed a trend reported in hospitalized children that showed a higher level in organization [38]. Future studies could address the hypothesis that T1DM might deal with distressing events related to their illness through a more sophisticated cognitive effort in their narrative, trying to prevent the outbreak of painful emotions. Focusing on coping strategies, results showed that CF children differ from the normative sample in the use of problem-focused coping strategies. Children appeared to be less able to include cognitive decision-making and direct problem-solving in the management of disease-related stress. In line with Denford and colleagues [26], patients affected by chronic, non-curable conditions, could use problem-solving maneuvers less frequently. Children with T1DM and leukemia used less distraction strategies, in respect to children of the normative sample, in line with evidence reported by Delvecchio and colleagues [38] with hospitalized children. Presumably, T1DM and leukemic children are critically exposed to invasive medical procedures, and they may show some difficulties in applying distraction behaviors due to the overstimulating nature of these procedures. Positive coping strategies, which include distraction of attention, imagery, and play, probably need to be solicited in young children by adults. Parents can be supportive in allowing their child to use distraction strategies, using, for example, their favorite toys or games during day hospital or hospitalization periods. According to the comparison between T1DM, CF, and Leukemia, no differences emerged across the illnesses in respect to coping strategies. Although sub-samples are small and do not grant generalizations, presumably, T1DM, CF, and Leukemic children share challenges in coping with chronic illness. Moreover, literature suggested that coping strategies might depend on environmental and family factors, such a financial situation [21]. The present paper did not focus on those variables, but all three clinical subgroups belong to the middle-class, sharing similar contextual resources. Future investigations need to be applied in order to deepen contextual and family issues. 

In regards to the second aim, T1DM and CF children showed a higher score in the total amount of reported psychosocial symptoms in comparison to the normative sample. Current literature supports this evidence, suggesting that chronically ill children are more prone to show impairment in psychological functioning. Moreover, evidence of the present study confirmed higher levels of perceived difficulties with peers in children with T1DM and CF, consistent with literature [11,12]. Prolonged separation from school and friends, typical with chronically ill children, might lead to feelings of social exclusion [8]. Furthermore, T1DM and CF patients reported higher levels of perceived difficulties in the scale of hyperactivity/inattention. Literature confirms that CF and T1DM children frequently suffer from attention deficit hyperactivity disorder (ADHD) [15]. Although none of the children enrolled in the present study presented a clinical diagnosis of ADHD, patients reported perceived symptoms related to hyperactivity inattention. Specifically regarding T1DM, a study by Shinosaki and colleagues [13] found indicators of inattention related to hyperglycemia and impulsivity with hypoglycemia episodes. Chronic inflammation that characterized CF lung disease may trigger ADHD symptoms in susceptible patients [14]. Moreover, T1DM children perceived a great number of prosocial attitudes in respect to healthy children. The presence of this important relational resource could mitigate the effect of difficulties with peers and, broadly speaking, could be considered a protective factor for T1DM psychological functioning.

Furthermore, T1DM and CF scored higher in the presence of separation anxiety symptoms than normative sample peers. Consistently with literature, pediatric chronic patients are at significant risk for the development of anxiety disorders [10]. Due to their disease management including day hospital or hospitalization periods, they could experience worry and distress about being away from their parents [20]. Regarding the comparison between T1DM and CF, CF children reported higher symptoms of separation anxiety than T1DM. CF is a medical condition diagnosed from pregnancy or birth. CF children might have experienced early life distress, such as frequent early separation from caregivers that may coincide with stressful and painful investigations and with the risk of death. This early life adversity might heighten developmental vulnerability to separation anxiety symptoms for CF children. 

Finally, in regards to the third aim, in the T1DM sample, a positive affective tone in the story and a good ability in creating a coherent and well-integrated APS-Br narrative corresponded with higher use of the ability to cope with distressful events through positive cognitive restructuring skills. Evidence suggested that in the field of pediatric chronic illnesses, the ability to positively restructure unpredictable events displayed adaptive efforts to stressors related to the illness itself [23]. Cognitive restructuring seemed to be linked with greater wellbeing in pediatric diabetes [27], presumably indicated by a general adaptive play in T1DM sample of the present study. Results highlighted also association between pretend play and symptoms. T1DM children showed a positive correlation between the number of affects named in their play stories and the total amount of symptoms self-reported. Future investigation needs to be carried out to spread light on this evidence. APS-Br scoring system indicated just a total score of affects expressed in the play stories, with no distinction between positive and negative. Anyway, it seems that the more T1DM children expressed affective themes in the 5 min tasks, the more they reported also negative psychological states. Future research needs to clarify the nature of this evidence. On one hand, it might be possible that TD1M children express, in the play narrative, emotion such as sadness, frustration, and anger, maybe associated to management of their chronic condition. This could explain the association with the perceived symptomatology and highlight T1DM children’s willingness in expressing through play painful emotions associated with the distress that their chronic condition could lead to [32]. This evidence supports the idea that play might facilitate exploration and elaboration of painful experiences, enhancing the integration between internal states and behaviors [33]. In respect to CF samples, the positive emotional climate of the story is associated with prosocial resources, suggesting that an adaptive emotional atmosphere during the play session is associated with psychological resources in the relational contest, important skills in CF children who present relational difficulties. 

Several limitations of the present study need to be taken into account when interpreting the results. First, no healthy children were enrolled to constitute the control group, but comparison with normative samples was carried out. Although no study included three different chronic samples, sample size was small and results might not be generalizable. The absence of contextual and family factors assessment might be a limitation for interpreting some results. The introduction of a multiple informant method may be a benefit for future studies, in order to better understand how children and families cope with medical chronic illnesses. 

## 5. Conclusions

Through a cross-illness study, this paper highlighted the functioning of chronically ill children, focusing on pretend play, psychological functioning, and coping strategy assessments. Pretend play seemed to be a useful way to express and explore both positive and negative emotions. Few studies investigated the role of pretend play as an assessment measure, but more frequently as intervention in chronic population. Baseline assessment is important, as well, in medical contest, in order to handle or prevent anxiety from worsening, improve self-esteem, and improve medical regimen compliance [34]. Then, non-invasive, and easy applicability of pretend play and self-reporting could help medical professionals sustain children from an affective perspective during visits or hospitalization episodes. With application of a multimethod approach, clinicians can intercept early child weaknesses and, consequently, direct the interventions specifically towards the development of alternative and more adequate developmental trajectory. An assessment protocol, as proposed in the paper, could also be repeated during time and hospital admission, in order to monitor changes in coping strategies, symptoms, and skills in expressing emotions. Good psychological functioning might be considered as a protective factor, enhancing compliance in following medical protocols requested for the diseases. 

## Figures and Tables

**Table 1:** Cohen’s *d* (effect size) for type 1 diabetes mellitus (T1DM), cystic fibrosis (CF), and leukemic children in comparison with normative samples for Affect in Play Scale Brief Version (APS-Br) and Children’s Coping Strategies Checklist–Revision1 (CCSC-R1).

	T1DM (*n* = 16)			CF (*n* = 13)			Leukemia (*n* = 15)		
APS-Br	M	SD	*d*	M	SD	*D*	M	SD	*d*
Organization	3.13	1.54	0.614 **	2.85	1.57	0.391 *	1.73	0.96	−0.638 **
Imagination	2.44	0.96	0.073	2.23	1.01	−0.147	1.53	0.92	−0.932 ^§^
Comfort	3.19	0.91	0.021	2.62	1.19	−0.548 **	2.20	1.08	−1.024 ^§^
Frequency of affect expression	3.69	0.70	0.064	3.23	1.36	−0.370 *	2.73	1.16	−0.919 ^§^
Tone	2.63	0.62	−0.388 *	2.62	1.04	−0.316 *	2.07	0.88	−0.968 ^§^
**CCSC-R1**									
Problem-Focused Coping	2.37	0.67	−0.459 *	2.23	0.62	−0.731 ^**^	2.71	0.65	0.114
Positive Cognitive Restructuring	2.29	0.52	−0.462 *	2.33	0.47	−0.396 *	2.51	0.61	−0.008
Distraction Strategies	2.55	0.54	−0.607 **	2.68	0.45	−0.388 *	2.57	0.51	−0.574 **
Avoidance Strategies	2.38	0.42	−0.203 *	2.39	0.60	−0.165	2.55	0.49	0.081
Support-Seeking Strategies	2.38	0.58	0.20 *	2.31	0.58	0.278 *	1.91	0.70	−0.386 *

* small effect; ** medium effect; ^§^ large effect [53].

**Table 2:** Cohen’s d for T1DM and CF groups in comparison with normative sample.

	T1DM (*n* = 16)			CF (*n* = 13)		
SDQ	M	SD	*d*	M	SD	*d*
Total Difficulties Score	19.69	3.91	0.991 ^§^	18.54	5.16	0.673 **
Emotional Symptoms	5.00	2.85	0.420 *	4.46	2.44	0.229 *
Conduct Problems	3.50	1.37	0.060	3.46	1.71	0.034
Hyperactivity-Inattention	5.75	1.69	0.726 **	5.69	1.97	0.656 **
Peer Problems	5.44	1.50	1.283 ^§^	4.92	1.19	1.058 ^§^
Prosocial Behavior	8.25	2.49	0.691 **	7.23	2.31	0.261 *
**SASI-C**						
Total Score	20.63	8.07	0.608 **	35.46	7.90	2.385 ^§^
Separation Anxiety	11.56	4.05	0.560 **	16.56	4.67	1.508 ^§^
Fears of something that can happen in the night	4.13	3.18	0.304 *	9.38	3.01	2.075 ^§^

* small effect; ** medium effect; ^§^ large effect [53].

**Table 3:** Mann–Whitney U test for SASI-C variables between T1DM and CF groups.

	T1DM (*n* = 16)		CF (*n* = 13)		*p*
SASI-C	M	SD	M	SD	
Separation Anxiety	11.56	4.05	16.54	4.67	0.007
Fears of something that can happen in the night	4.13	3.18	9.38	3.01	0.000
Total	20.63	8.07	35.46	7.90	0.000
